# miRNA-26b Is Associated with Increased Connexin-40 Expression in Endothelial Cells Under Flow Conditions

**DOI:** 10.3390/ijms27104644

**Published:** 2026-05-21

**Authors:** Marcus Igl, Markus Haberbosch, Michael Hristov, Felix Reich, Emiel P. C. van der Vorst, Christian Weber, Kiril Bidzhekov

**Affiliations:** 1 Institute for Cardiovascular Prevention (IPEK), Ludwig-Maximilians-Universität (LMU) München, 80802 Munich, Germany; marcus.igl@uni-tuebingen.de (M.I.); markus.haberbosch@med.uni-muenchen.de (M.H.); michael.hristov@med.uni-muenchen.de (M.H.); felix.reich@praxisservice.org (F.R.); evandervorst@ukaachen.de (E.P.C.v.d.V.); 2 Department of Internal Medicine I, Aachen-Maastricht Institute for Cardio-Renal Disease (AMICARE), Institute for Molecular Cardiovascular Research (IMCAR), University Hospital Aachen, RWTH Aachen University, 52074 Aachen, Germany

**Keywords:** connexins, Cx40, micro-RNA, miRNA-26b, atherosclerosis

## Abstract

Endothelial cell dysfunction is the initial step in atherosclerosis, in which gap junction proteins such as connexin 40 (Cx40) might play an important role. Previously, we could demonstrate that miRNA-26b, a 21-nucleotide miRNA, is highly expressed in human atherosclerotic plaques and plays a key causal role in atherogenesis. There is evidence that miRNA-26b and Cx40 play crucial roles in sustaining endothelial health. However, their potential effects on atherosclerosis-related processes remain poorly understood. Therefore, this study elucidated the expression of miRNA-26b and Cx40 and studied the effect of Cx40 on inflammation and monocyte binding, which are key processes in atherosclerosis formation. In a human in vitro endothelial cell model, miRNA-26b overexpression is associated with increased Cx40 expression. Although we did not observe any anti-atherogenic effect of Cx40 on monocyte attachment or VCAM-1 transcription under static conditions, a flow-dependent expression pattern characterised by increased Cx40 and reduced VCAM-1 transcription was observed. How miRNA-26b and Cx40 are connected remains to be investigated. Furthermore, the functional role of Cx40 under flow conditions requires further investigation.

## 1. Introduction

Atherosclerosis is an inflammatory disease initiated by endothelial injury, during which multiple events are triggered. For example, expression of vascular cell adhesion molecule-1 (VCAM-1) is instrumental in leukocyte binding to the endothelial layer. This initiates the invasion of monocytes into the tunica intima, where they transform into macrophages and then into foam cells [1,2]. Due to excessive lipid uptake, these foam cells eventually die, starting the formation of the necrotic core and the further progression of atherosclerotic plaques [3]. In our study, we aimed to identify new key players implicated in endothelial dysfunction.

The 21st century ushered in a golden age for microRNA (miRNA) research [4]. Although miRNAs were initially found in C. elegans, there are already over 3000 different miRNAs discovered and described in mammals [4,5]. Several of these are linked to endothelial activation and inflammation, including miRNA-26b, which is overexpressed in human atherosclerotic plaques [6]. Importantly, our recent findings demonstrated that miRNA-26b deficiency, in particular, and non-haematopoietic miRNA-26b deficiency significantly augment atherosclerosis, by increasing endothelial inflammation and VCAM-1-dependent leukocyte adhesion [7,8], highlighting a functional connection between miRNA-26b and endothelial inflammatory pathways [7]. To evaluate the effect of miRNA-26b in vivo, a mouse knockout model was established, and transcriptome analysis revealed differential gene expression compared to wild-type controls [6]. One altered gene was the Gap junction alpha-5 gene (Gja-5). The transcription profile of Gja-5, belonging to the GJ family, is strongly decreased in *miRNA-26b^−/−^* mice in comparison to wild type [6]. The GJ family of genes encodes the connexin protein family, including Gja-5, such as connexin 40 (Cx40) [9]. Connexins consist of four transmembrane domains, two extracellular loops and three cytoplasmic regions [10], and they form a hexamer with a small channel in the middle, which is called a connexon [11]. Connexons of two cells form so-called gap junctions by the interaction of the extracellular loops of their connexins [12], providing the transport of metabolites like Ca^2+^, ATP, and even miRNA [13,14]. These small molecules enable the communication between two different cells [15]. It is known that endothelial cells express Cx40, Cx37, and Cx43 [16]. A mouse model deficient for Cx40 in endothelial cells shows enhanced atherosclerosis compared with the wild type, coinciding with increased VCAM-1 expression [17]. VCAM-1 is activated through the NFκB pathway, which marks a central pathway of inflammation in endothelial cells [1,18]. Although most of these effects are well studied in mice, evidence in humans is rather limited.

Therefore, we analysed the impact of miRNA-26b overexpression on Cx40 transcription and expression in human endothelial cells in vitro (human umbilical vein endothelial cells; HUVECs). We observed the effect of Cx40 on VCAM-1 transcription and monocyte binding under static conditions. Moreover, the mRNA transcription of Cx40 and VCAM-1 was also measured under flow conditions. Lastly, we confirmed that overexpressing miRNA-26b in human aortic endothelial cells (HAoECs) also increases Cx40 expression.

## 2. Results

### 2.1. Cx40 Is Upregulated in HUVECs upon miRNA-26b Overexpression

**Figure 1:**
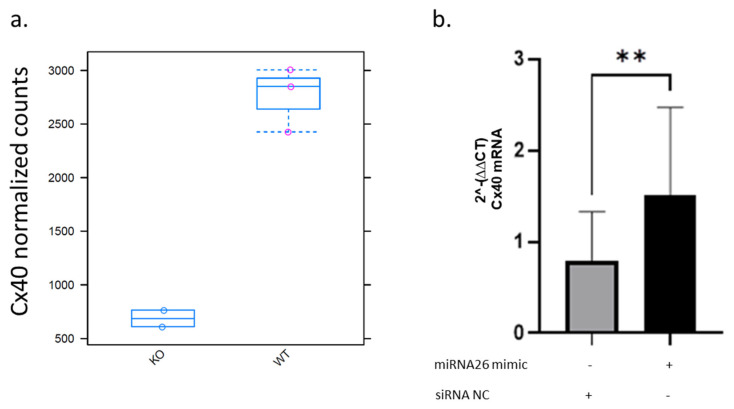
Cx40 transcription levels under miRNA-26b modulation in mouse and human endothelial cells. (**a**) Cx40 mRNA counts in thoracic-abdominal aortic tissue comparing wild-type (WT) and miR-26b knockout (KO) mice. (**b**) Cx40 mRNA transcription in HUVECs transfected with miRNA-26b mimic compared to non-coding control (siNC). Data are presented as mean ± SD. *n* = four independent biological replicates; each experiment included technical duplicates/triplicates. Statistical analysis was performed using an unpaired Student’s *t*-test. *p* < 0.01 = **.

Transcriptome analysis of thoracic-abdominal aortic vessels from *miRNA-26b^−/−^* mice in comparison to wild-type mice revealed multiple changes in their mRNA transcription [19]. For example, *Cx40* has a median count level of 600 in *miRNA-26b^−/−^* mice, while wild-type mice have a median value of 2800 counts, demonstrating that the lack of miRNA-26b results in a striking 78% decrease in *Cx40* transcription (Figure 1a). These effects could also be validated in HUVECs in vitro, as cells overexpressing miRNA-26b show a highly significant (*p* = 0.0022) twofold induction of *Cx40* transcription, compared to cells transfected with a non-coding siRNA (siNC) (Figure 1b). These data support an association between miRNA-26b expression and Cx40 levels.

### 2.2. VCAM-1 Expression Is Independent of Cx40 Protein Level Under Static Conditions

**Figure 2:**
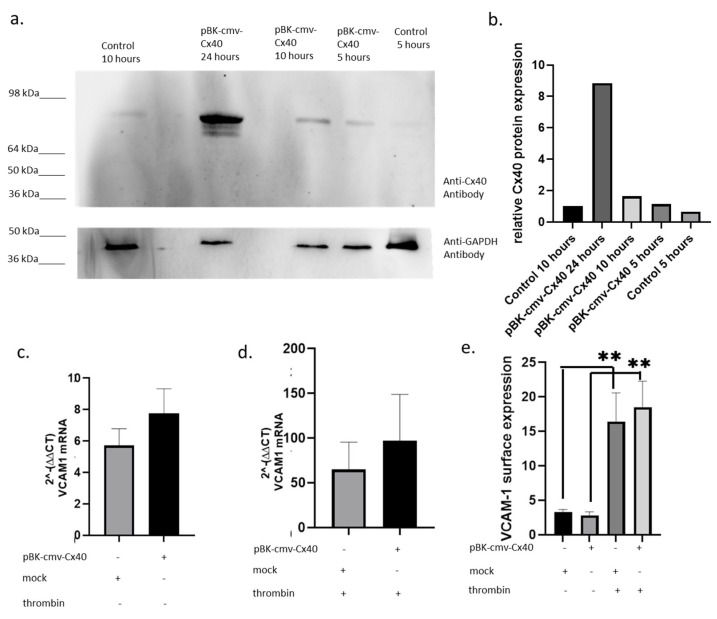
Effect of Cx40 overexpression on VCAM-1 transcription and expression in HUVECs. (**a**) Representative Western blot of Cx40 expression following transfection with pBK-cmv-Cx40. (**b**) Densitometric analysis of Cx40 protein levels at indicated time points (5, 10, and 24 h), normalised to GAPDH. (**c**) VCAM-1 mRNA transcription in HUVECs transfected with pBK-cmv-Cx40 compared to mock control. (**d**) VCAM-1 mRNA transcription following thrombin stimulation in Cx40-overexpressing and control cells. (**e**) Surface expression of VCAM-1 assessed by flow cytometry. Data are presented as mean ± SD; *n* = 4 independent biological replicates. Statistical analysis was performed using an unpaired Student’s *t*-test. *p* < 0.01 = **.

Cx40 has an anti-atherosclerotic function in mice as it can inhibit the NFκB pathway and VCAM-1 expression [17,20]. Therefore, it is of interest to analyse whether Cx40 has a similar effect in human conditions. To investigate the potential role of Cx40 in VCAM-1 expression, a vector with Cytomegalovirus promoter (cmv) was cloned to drive the overexpression of human Cx40 in HUVECs (pBK-cmv-Cx40; Figure 2a). Within the first 10 h after transfection, Cx40 expression was 64% higher than in the control. After 24 h, the Cx40 expression was seven times higher than that of the control (Figure 2b). To stimulate NFκB pathway induction, thrombin was used, as unlike TNF-α, it did not affect Cx40 expression [21,22]. Overexpression of Cx40 induced a non-significant 35% higher VCAM-1 mRNA transcription in comparison to mock-transfected HUVECs (Figure 2c). In the thrombin-treated samples, a similar tendency was observed (Figure 2d). In addition, we analysed VCAM-1 expression on the surface of HUVECs (Figure 2e). Interestingly, Cx40 upregulation was associated with a slight downregulation of VCAM-1 compared to mock transfection. In contrast to this observation, samples which were treated with thrombin show slightly higher, though non-significant, VCAM-1 expression when Cx40 is overexpressed (Figure 2e). Therefore, our results provide no evidence that human Cx40 affects VCAM-1 expression under static conditions.

### 2.3. Monocyte Binding Is Not Influenced by Modulation of Cx40 Overexpression in Static Conditions

**Figure 3:**
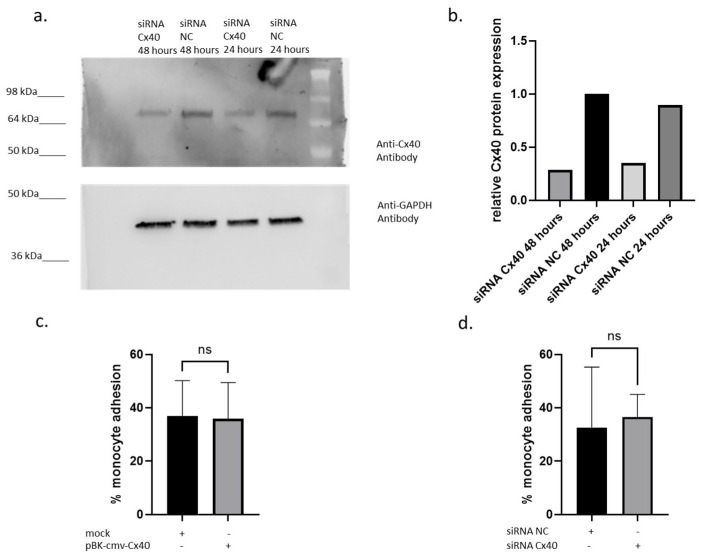
Cx40-dependent binding ability of monocytes to endothelial cells. (**a**) Western blot and (**b**) densitometry analysis of Cx40 in siRNA Cx40 and siRNA NC-treated HUVECs, respectively, after 24 and 48 h. (**c**) Comparison of the monocyte endothelial binding between pBK-cmv-Cx40 and mock (*n* = five independent biological, three technical replicates). (**d**) Difference in the monocyte endothelial binding in siRNA NC and siRNA Cx40 HUVECs (*n* = three independent biological, four technical replicates). All *p*-values are calculated with unpaired Student’s *t*-test. ns = not significant.

A crucial step in atherosclerotic progression is monocyte adhesion to endothelial cells at sites of inflammation. Therefore, the effects of Cx40 overexpression on monocyte adhesion were analysed using a monocyte adhesion assay based on Vincent et al. [23]. It could be observed that Cx40 was clearly downregulated in samples treated with siRNA targeting Cx40 in comparison to control siRNA (NC)-treated samples (Figure 3a). Indeed, a 61% decrease in Cx40 protein expression was observed after 24 h, and up to 72% after 48 h (Figure 3b). On the other hand, Cx40 overexpression did not affect monocyte adhesion compared with mock transfection (Figure 3c). Accordingly, siRNA Cx40 also demonstrated non-significant monocyte binding compared to siRNA NC control samples (Figure 3d). Thus, this functional test could reveal that Cx40 does not affect monocyte endothelial binding, indicating that Cx40 does not exhibit anti-inflammatory or anti-atherogenic functions in endothelial cells under static conditions.

### 2.4. Cx40 mRNA Is Upregulated While VCAM-1 Is Downregulated Under Flow Conditions

**Figure 4:**
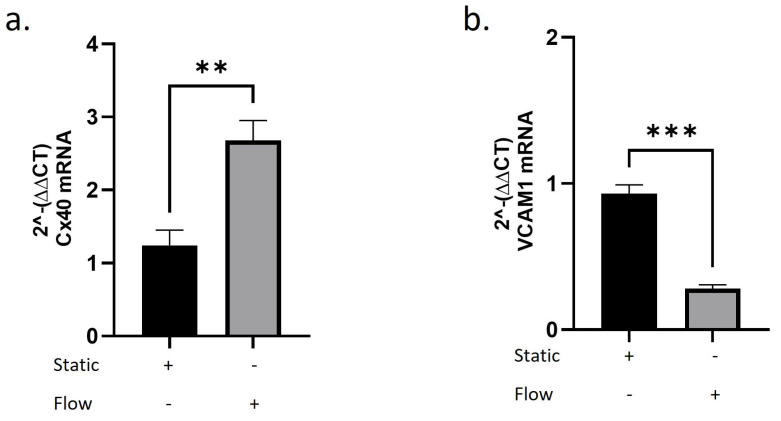
mRNA transcription of Cx40 and VCAM-1 in HUVECs under static and flow conditions. HUVECs were exposed to laminar shear stress (12 dyn/cm^2^) for 72 h using an ibidi flow system. Static cultures served as controls. (**a**) Cx40 mRNA transcription normalised to GAPDH under static and flow conditions. (**b**) VCAM-1 mRNA transcription normalised to GAPDH under static and flow conditions. RNA was isolated from parallel samples and analysed by qPCR. Data are presented as mean ± SD; *n* = three independent biological replicates. Statistical analysis was performed using unpaired Student’s *t*-test. *p* < 0.01 = **; *p* < 0.001 = ***.

A recent study reports that HUVECs increase Cx40 expression under both high and low shear stress, but not under oscillatory conditions [18]. It is of interest to investigate how Cx40 and VCAM-1 transcription changes under shear stress. Therefore, our next experimental approach was to examine VCAM-1 and Cx40 mRNA transcription in HUVECs under static conditions (static) and under flow conditions (flow). We applied a moderate shear stress of 12 dyn/cm^2^ and observed a twofold upregulation of Cx40 mRNA transcription under flow conditions (Figure 4a). In contrast, VCAM-1 mRNA transcription was markedly reduced compared to static conditions (Figure 4b). These findings indicate a flow-associated transcription pattern characterised by increased Cx40 and decreased VCAM-1 mRNA levels.

### 2.5. Validation of Cx40 Modulation in Human Arterial Endothelial Cells (HAoECs)

**Figure 5:**
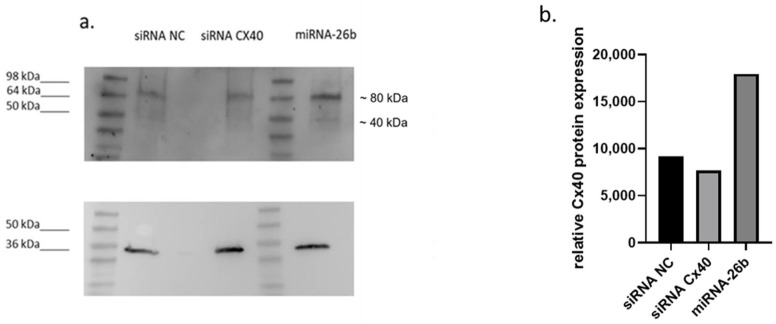
Validation of Cx40 modulation in human arterial endothelial cells (HAoECs). (**a**) Representative Western blot showing Cx40 protein levels in HAoECs following transfection with control siRNA (siRNA NC), siRNA targeting GJA5 (siGJA5), and miRNA-26b. GAPDH was used as a loading control. (**b**) Densitometric analysis of Cx40 protein levels normalised to GAPDH and expressed relative to control (siRNA NC). Quantification is shown from a representative experiment.

To address the relevance of our findings in arterial endothelial cells, we performed additional experiments in human arterial endothelial cells. Consistent with our observations in HUVECs, modulation of Cx40 expression was detectable at the protein level (Figure 5a). In particular, siRNA-mediated knockdown of GJA5 reduced Cx40 protein levels, whereas miRNA-26b overexpression increased Cx40 signal compared to control conditions. The detected band migrated at a higher apparent molecular weight (~80 kDa), consistent with previously described connexin oligomerisation. In addition to the predominant higher-molecular-weight signal, we observed a weaker band at the expected monomeric size of approximately 40 kDa, particularly visible in Figure 5a. However, under our experimental conditions, the majority of the detectable Cx40 signal appeared in the higher-molecular-weight form. Increasing protein loading to further enhance the monomeric signal substantially reduced blot quality and impaired reliable densitometric quantification (Figure 5b).

These findings indicate that modulation of Cx40 expression is not restricted to venous endothelial cells but can also be observed in arterial endothelial cells.

## 3. Discussion

In our previous work, we provided the first evidence that miRNA-26b contributes to atherosclerosis, as it is overexpressed in human atherosclerotic plaques [6], whereas miRNA-26b deficiency results in increased atherosclerosis in mice [7,8]. Atherosclerosis is characterised by permanent inflammation [24]. In recent years, numerous studies have reported the protective role of Cx40 in atherosclerosis [20,25,26]. On the other hand, several studies highlight that miRNA-26b plays a potent role in regulating inflammation; for example, platelet-derived growth factor β (PDGFRβ) is a highly likely downstream target of miRNA-26b that affects mouse liver fibrosis and angiogenesis [8]. Additionally, Ge et al. [27] demonstrated in mice that the pro-inflammatory prostaglandin–endoperoxidase synthase 2 (PTGS2) is also a target of miRNA-26b. Furthermore, miRNA-26b and NS398 can reduce TNF-α and IL-6 expression and increase IL-10 levels, and there is evidence that miRNA-26b interacts with IL-6 in murine microglia [28]. Recently, we demonstrated in our miRNA-26b knockout mouse model that Cx40 is downregulated compared with wild-type animals [19]. Consistent with our findings, Calderon et al. [14] reported in their review that Cx40 is associated with miRNA-26b. However, the exact correlation between these two players was not clarified, nor was the effect of the miRNA-26b-Cx40 interaction on atherosclerosis. Our data suggest that miR-26b contributes to the regulation of Cx40 expression under flow conditions. While the precise mechanism remains unclear, microRNAs can, in certain contexts, regulate gene expression through non-canonical mechanisms, including transcriptional activation. However, such mechanisms have not been demonstrated for miR-26b in the present system. Interestingly, in vitro, we confirmed that human Cx40 is upregulated in HUVECs treated with miRNA-26b mimic. This would give evidence that miRNA-26b is associated with increased Cx40 expression. However, this can also be caused by other factors, indirectly linking miRNA-26b and Cx40. Further studies will be required to determine the mechanism which is behind this. Because the function of human Cx40 in atherosclerosis remains poorly understood, we investigated it in vitro in HUVECs. The observed band at approximately 80 kDa may reflect dimerisation or higher-order connexin structures (oligomerisation) and phosphorylation, which have been described for connexin proteins [29], although antibody specificity and protein conformation cannot be excluded. Dimerisation was already seen in Cx43; as Cx40 has a similar helical structure, it is expected to behave similarly [30]. Importantly, in addition to the predominant signal detected at approximately 80 kDa, we observed a weaker band at the expected monomeric molecular weight of ~40 kDa, supporting the detection of Cx40 protein in our system. Under our experimental conditions, however, most of the detectable signal appeared in the higher-molecular-weight form, which may reflect dimerisation, oligomerisation, or post-translational modifications of connexin proteins. Attempts to increase protein loading to further enhance the monomeric signal resulted in reduced Western blot resolution and compromised the reliability of subsequent densitometric analyses. VCAM-1 binds to α4β1 integrin on monocytes, highlighting its crucial role in atherosclerosis [8,31]. Cx40 overexpressing HUVECs mostly showed higher VCAM-1 transcription and expression with and without thrombin treatment in comparison to mock-transfected HUVECs. This contrasts with the findings of Chadjichristos et al. [17] in mice with an endothelial-specific deletion of Cx40. In the monocyte adhesion assay, higher binding efficiency correlated with decreased Cx40 expression. These observations are in line with previously reported protective roles of Cx40 in murine models, indicated by Denis et al. [20]. However, our data in human endothelial cells do not confirm such a role under static conditions; we did not observe a significant effect on VCAM-1 transcription or on monocyte binding. Vorderwülbecke et al. [32] already showed that Cx40 expression is enhanced under high shear stress. Therefore, phenotypic changes in endothelial cells may only become apparent under flow conditions. We could provide the first evidence of upregulation of Cx40 mRNA and a parallel downregulation of VCAM-1 mRNA under flow. Importantly, our data do not demonstrate a causal role for Cx40 in regulating VCAM-1 transcription or monocyte adhesion in human endothelial cells. While previous studies in murine models have suggested an anti-inflammatory role of Cx40, our in vitro findings under static conditions did not confirm such effects. Moreover, under flow conditions, we observed a coordinated transcription pattern characterised by increased Cx40 and decreased VCAM-1 mRNA levels. However, these observations are based on parallel measurements and do not establish a direct mechanistic link between Cx40 and VCAM-1 regulation. Additional loss-of-function and rescue experiments will be required to determine whether Cx40 contributes functionally to endothelial responses under shear stress. Furthermore, it has to be shown whether miRNA-26b contributes to the molecular pathway underlying this interaction.

To strengthen the translational relevance of our findings, we performed additional validation experiments in HAoECs. miRNA-26b overexpression indicated the modulation of Cx40 protein levels, as observed and confirmed by densitometric analysis of a representative experiment. While these data strengthen the generalizability of our findings, they remain descriptive and do not provide direct mechanistic insight.

In summary, our study demonstrates that miRNA-26b is associated with increased Cx40 expression in human endothelial cells. While Cx40 did not influence endothelial activation under static conditions, a flow-dependent expression pattern characterised by increased Cx40 and reduced VCAM-1 was observed. These findings suggest that Cx40 may be part of a flow-responsive endothelial programme relevant to vascular homeostasis. However, the underlying molecular mechanisms and the functional role of Cx40 in this context remain to be determined.

## 4. Material and Methods

### 4.1. Cloning

To gain the template for the *pBK-cmv-Cx40* vector HUVEC mRNA was isolated and transcribed into cDNA. Using the primer pair GJA-5 forward primer (ATGGGCGATTGGAGCTTCC) and GJA-5 reverse primer (TCACACTGATAGGTCATCTGAC) targeting the GJA-5 cDNA a preliminary insert was amplified. The primer pair GJA-5 forward (CTGCAGATCCGCTAGATGGGCGATTGGAGCTTCC) and GJA-5 reverse (AGTAATTGCGGCTAGTCACACTGATAGGTCATCTGAC) for In-Fusion were designed with the Cloning Primer design tool (Legacy In-Fusion Primer Design Tool (HTML/JavaScript web utility) from Takara. For the construction of the vector, a NEBuilder^®^ Hifi DNA Assembly cloning Kit (New England Biolabs, Ipswich, MA, USA) used according to its protocol. A vector insert ratio of 1:2 was applied with *pBK-cmv* as backbone. A total of 2 µL of the reaction was brought onto Stellar competent bacteria. Briefly, they were kept on ice for 10 min, then heat shocked at 42 °C for 30 s. Subsequently, the transformants were kept on ice for 2 min, and 950 µL of SOC medium was added. After 60 min of incubation at 37 °C. Coli were plated on LB_Amp_ plates. Subsequently, the colonies containing the correct insert were screened by colony PCR.

### 4.2. Cell Culture

HUVECs were cultivated from passage 5 to 7 in endothelial cell medium (Promocell, Heidelberg, Germany) supplemented with 1:1000 Gentamycin and split every 4 to 5 days. Thrombin treatment of HUVECs was performed at 5 U/mL. THP-1 cells were grown in RPMI-1640 supplemented with 1% Sodium Pyruvate, 1% Pen/Strep, and 1% Glutamine. They were split every 5 days. The HAoECs (Promocell, C-12271) were cultivated in a similar way. Briefly, they were kept from passage 5 to 7 in endothelial cell medium specific for HAoECs (Promocell, C-22211) with 1:1000 Gentamycin. The cells were passaged every 4 to 5 days.

### 4.3. Transfection

8 × 10^5^ HUVECs or HAoECs were resuspended in 100 µL of Lonza transfection media from the Amaxa^TM^ HUVEC Nucleofector^TM^ Kit. The transfection media were supplemented, respectively, with 4 µg of *pBK-cmv* vector, 4 µg *pBK-cmv-Cx40* vector, 30 pmol siRNA against Cx40 (GJA-5-HSS104129, Thermo Fisher Scientific, Waltham, MA, USA), 30 pmol siRNA NC (1027281, Qiagen), and 30 pmol miRNA-26b (MISSION^®^ micro-RNA, Sigma Aldrich, St. Louis, MO, USA). Electroporation was performed with the Amaxa Nucleofector^TM^ (programme A-34). The transfected HUVECs or HAoECs were seeded and incubated for 2 h in endothelial cell media, then the medium was exchanged with fresh medium.

### 4.4. Monocyte Adhesion Assay and Flow Cytometry

The monocyte adhesion assay was performed as described by Vincent et al. [23]. Briefly, THP-1 cells were incubated in a working concentration of 5 ng/mL DiI for 30 min (Thermo Fisher Scientific). HUVECs were incubated with the stained THP-1 cells for 30 min. Subsequently, the cells were washed 3× with PBS, resuspended in Accutase, and then in 400 µL PBS. For VCAM-1 surface expression, HUVECs were dissolved by Accutase and washed with 1% BSA. Following that, we incubated the HUVECs with 1% BSA with 1:100 Anti VCAM-1 Antibody (sc-13160 PE, Santa Cruz, CA, USA) for 30 min at 4 °C. The cells were washed twice with 1% BSA, then they were collected into 400 µL 1% BSA. Next, a BD FACS Canto II Flow cytometry assay was performed. The cell mix was analysed using FACS Diva Software 8.0.1 (Becton Dickinson, Franklin Lakes, NJ, USA) for THP-1 cell binding or VCAM-1 surface expression.

### 4.5. RNA Isolation, Reverse Transcription and qPCR

RNA isolation was performed according to the RNeasy kit protocol (Qiagen). The MMLV Reverse Transcriptase (Promega, Fitchburg, WI, USA) was used for the transcription of 1 µg RNA for 1 h at 37 °C into cDNA. The qPCR primer VCAM1 (Assay ID Hs01003372_m1, Thermo Fisher Scientific), Cx40 (Assay ID Hs979198_m1, Thermo Fisher Scientific) and GAPDH (Assay ID Hs03929097_g1, Thermo Fisher Scientific) were used for the Taq Man assay (Taq Man^TM^ Gene Expression Master Mix, Thermo Fisher scientific). The following temperature setting was applied for the qPCR, 50 °C 2 min, 95 °C 10 min, then 40 cycles of 95 °C 10 s and 60 °C 1 min.

### 4.6. Western Blot

The protein fractions from HUVECs or HAoECs were isolated with Cyto Buster and diluted with 2x Laemmli Buffer. Subsequently, equal amounts of 8.5 µg or 10 µg of protein were diluted with 2x Laemmli Buffer with 0.5 M of DTT. The samples were loaded into the pockets of a Mini PROTEAN TGX (12%) gel. A gelelectrophoresis and wet blot were performed. In the following, the membrane was blocked with blocking buffer (0.1% Milk powder) for 2 h. The primary antibody anti human Cx40 (ab183648, Abcam, Cambridge, UK) or anti GAPDH (MAB374) diluted 1:1000 was incubated overnight. After washing the membrane three times for 5 min with TBST, it was left for 2 h with the secondary antibodies anti rabbit (Sc-2004, Santa Cruz, CA, USA) with a concentration of 1:2000 and anti-mouse (HAF007, R & D Systems, Minneapolis, MN, USA) with a concentration of 1: 1000. Densitometry analysis was performed with ImageJ (Version 1.54r).

### 4.7. Flow Assay

Two µ-Slides I^0.4^ (Ibidi) were incubated for 1 day at 37 °C with 5% CO_2_. HUVECs were seeded to 1 × 10^6^ in 100 µL into the ibidi slides. After 30 min, 60 µL of medium was added to both reservoirs of the slides. After 4 h the perfusion system was set up according to the manual from ibidi. Briefly, the fluidic unit (ibidi) with 6 mL of media in both syringes and one 1 mL of residual media was connected to the ibidi pump system. After calibrating the system, we connected it with one of the ibidi slides for the dynamic system. The second slide was left unconnected as the static system. The following programme was initiated: cycle 2 dyn for 10 min then 12 dyn indefinite; the slides of the Fluidic unit were brought to 37 °C with 5% CO_2_. Next, after 3 days, the RNA of the static and dynamic system was isolated and a qPCR was performed.

### 4.8. miRNA-26b Knockout Mouse Model

miRNA-26b knockout (miRNA-26b^−^/^−^) mice were generated as previously described [19]. Briefly, the miR-26b genomic locus was deleted using homologous recombination. Mice were maintained under standard conditions, and genotyping was performed as described previously [19].

### 4.9. Statistic

The statistical analysis was performed with Graph Pad Prism 9 software (Graph Pad Inc., Boston, MA, USA). The means of two different groups were compared with unpaired Student’s *t*-test. Differences with *p* < 0.05 were considered significant and marked with *. Differences with *p* < 0.01were marked with **. The data is shown with the mean ± SD. Each experiment was performed independently at least three times unless otherwise stated; n represents independent biological replicates.

## Data Availability

The original contributions presented in this study are included in the article. Further inquiries can be directed to the corresponding author.
